# Combating Racism Through Research, Training, Practice, and Public Health Policies

**DOI:** 10.5888/pcd20.230167

**Published:** 2023-06-29

**Authors:** Jeffrey E. Hall, L. Ebony Boulware

**Affiliations:** 1 Office of Health Equity, Centers for Disease Control and Prevention, Atlanta, Georgia; 2 Wake Forest University School of Medicine, Winston-Salem, North Carolina; 3 Advocate Health, Winston-Salem, North Carolina

Racism is “a system [of power and oppression] of structuring opportunity and assigning value based on the social interpretation of how one looks (which is what we call “race”) that unfairly disadvantages some individuals and communities, unfairly advantages other individuals and communities, and saps the strength of the whole society through the waste of human resources” (1). At a systems level, racism is a public health problem, threat, and crisis (2–4). Its presence in society’s policies, practices, and programs creates inequities in access to vital conditions for health and well-being based on social ascriptions of race and ethnicity — resulting, for instance, in disparate access to and the quality of basic requirements for health and safety; residential neighborhood and housing options; developmental and educational experiences; and jobs, careers, and lifestyles (5–11). These inequities, in turn, produce and perpetuate disparities in health and disease experiences and outcomes. Because of its omnipresence, racism permeates every level of society, including the health care and public health sectors, creating racial and ethnic inequities in the operations of their infrastructures and, accordingly, in the delivery of essential services (12–21).

The work in this collection, “Combating Racism Through Research, Training, Practice, and Public Health Policies,” captures insights on roles and actions taken in public health, medicine, and policy to eliminate racism as a public health threat. *Preventing Chronic Disease* solicited manuscripts to elucidate research, training, practice, and public health policy–based efforts that address topics ranging from the effects of racism and racial discrimination on psychological, mental, and emotional health and disease risk to institutional, organizational, or community policies and changes implemented to address institutional racism. Articles in this collection 1) link exposures to racial discrimination with morbidity among diverse populations; 2) detail implementation of multicomponent antiracist initiatives enacted in schools of public health, schools of medicine, and other university-affiliated units; and 3) elevate attention to underlying drivers of structural inequities in housing and to domains through which meaningful community engagement in health initiatives is achievable.

## Racial Discrimination Experiences and Morbidity

The creation of racially and ethnically patterned differences in morbidity and mortality is well documented — covering many populations and health dimensions (7,22–26). However, continued expansion and updating of knowledge about how racism affects health, and who it affects, are critical to ensure that health care and public health remain capable of accounting for and mitigating the effects of all its manifestations. Original research by Reyes-Ortiz et al (27) demonstrates the continued salience of personally mediated racism and interpersonal racial discrimination as an emphasis toward which the performance of core functions must be directed and adapted.

Experiencing rejection, unfair treatment, or discrimination because of the meanings assigned to race, ethnicity, and skin color affects the odds of experiencing 2 or more chronic conditions concurrently in older adulthood among Colombians (27). Such experiences may increase or amplify the burden and complexity of multimorbidity patterns with which Columbian health care and public health systems must contend. They also may necessitate adoption of life course approaches to chronic disease management that are more socioecologically and clinically nuanced. Using racially informed, life course–anchored practice models may help assure equitable service delivery to older adults whose current health reflects culturally structured, race-related stress accumulated in social institutions during sensitive periods, developmentally significant social transitions, or ubiquitously over a lifetime (28–31).

## Implementation of Multicomponent Antiracist Initiatives in University-Affiliated Units

Academic organizations play an important role in perpetuating racism and its effects on health through institutional norms, pedagogy, and research practices (32–36). Several approaches to dismantling institutional racism are described in this collection. Rinderknecht et al (37) describe work to break down structurally racist processes and cultural barriers to entry into medical careers. They describe a novel longitudinal mentorship program for aspiring medical students who come from backgrounds underrepresented in medicine. Students articulated key areas they perceived as structurally racist barriers to successful medical school application, including difficulty with medical school entrance examination preparation, lack of mentorship, and financial considerations. Moreover, the authors describe a novel program in which racially and ethnically minoritized (hereinafter referred to as minoritized) medical students provide direct mentorship to aspiring racial and ethnic minority premedical students to help them overcome these barriers, resulting in increases in confidence and competencies required for successful medical school application. Their work provides a model for enhancing the entry of students from minoritized communities into medical and public health careers.

Both Allen et al (38) and Polston et al (39) describe efforts to eliminate institutional-level racism in schools of public health through organizational change efforts. Allen et al describe a comprehensive process at the University of California, Berkeley, whereby the organization is undergoing an active and transformative longitudinal process to embed antiracism throughout the school’s culture and practices. Efforts focus on multiple facets of the school’s community and culture, including improvement of faculty and workforce development, student experiences, curriculum and pedagogy, community outreach, and business processes. They describe robust efforts to collect data to drive assessment and accountability and provide an exemplar for similar efforts. Polston et al describe similarly motivated efforts at the University of North at Carolina Chapel Hill’s Gillings School of Global Public Health, whereby student activism and grassroots efforts, including qualitative data collection and analysis, led to the development of an institutional Equity Task Force. The task force developed and implemented antiracism actions in 6 areas, including 1) transforming culture and climate; 2) enhancing teaching, mentoring, and training; 3) revisiting how faculty and staff performance are assessed; 4) strengthening recruitment and retention of minoritized faculty; 5) increasing transparency in student hiring and resources; and 6) improving equity research–oriented planning. They provide a planning tool to help guide others in creating an antiracist institutional culture.

The approach to pedagogy in institutions of health-related higher learning also represents an important focus for antiracism efforts (17,34,40). Specifically, a need exists to ensure students in health-related fields are well trained to recognize and dismantle racism and to develop strategies to eliminate racism in their future professional practice. Durham Walker et al (41) describe a community heath course at Morehouse School of Medicine that trains medical students to work with minoritized and disadvantaged individuals and communities. This service-learning course shifts the lens of pedagogy beyond a traditional patient-centric focus on pathology to a diagnosis and assessment of the health of communities. Coursework helps students learn to develop action plans to improve aspects of community health, providing students with foundational knowledge of the effect of racism on health. This work provides a model for others seeking to fundamentally change workforce views on racism and its harmful effects on health, and to activate health professionals to dismantle the effect of racism on health through action.

Academic organizations also promote and support research that has promulgated well-earned distrust of medical research (42,43). Lebow-Skelley et al (44) acknowledge research centers as entities that affect faculty, students, and surrounding communities and have the potential to dismantle historically systemically racist research practices. They describe efforts at Emory University’s Rollins School of Public Health through the HERCULES environmental research program to 1) acknowledge and confront the university’s history of slavery and dispossession and 2) recognize and act on the need to address systemic and institutional racism in research practices. They embrace antiracist actions to transform their approach to university and academic partnerships with the ultimate goals of improving trust and accountability and creating equity in academic–community partnerships that provide a model for others.

## Drivers of Structural Inequities: Housing and Community Engagement

Health-based efforts to dismantle racism must eliminate racial and ethnic inequities in social determinants of health such as housing while maximizing community agency in health promotion and disease prevention (7–9,18,19,26,45). In her essay, Wonderly first encourages additional attention to housing as a particularly important social determinant of health. She links racial and ethnic disparities in COVID-19 risks and outcomes to inequities in housing access to further catalyze consideration of how housing costs, conditions, consistency, and contexts influence health, health care, and public health outcomes (46). Eliminating housing as a key arena where racial and ethnic health disparities are created requires interventions that expand and stabilize access to physically sound, high-quality, affordable housing in neighborhoods with robust environments, infrastructures, and institutions. Elevating humane housing as a vital condition for health as part of intersectoral action may aid in permanently expelling racism from this arena. Strategic integrations of Antiracist and Health and Equity in All Policies approaches could facilitate remediation of racist policies and practices that determine housing stock availability, neighborhood composition and resource allocations, and wealth accumulation opportunities associated with home ownership.

Although Wonderly’s discussion of housing calls for addressing features of social structure, her treatment of meaningful community engagement urges committed investment in enhancing community agency. At base, she asserts that meaningful advancement of health equity and systems transformation can result from strengthening partnerships and alliances, expanding co-created community knowledge, designing community-relevant health and health care programs and policies, and cultivating thriving communities (46). Centering and embracing historically marginalized racial and ethnic communities as true action partners via concerted investment in such domains may diminish power imbalances and reduce health disparities resulting from structural racism. Significant strides in dismantling and healing the harms of racist systems can be made together with communities who feel engaged and who capably wield tools for systems change in a manner consistent with their felt needs and interests.

## Conclusion and Directions Forward

The articles in this PCD collection provide inspiration for future efforts to dismantle racism in public health and medicine, and they also help identify gaps in the field for future progress. First, these articles demonstrate the need for continued efforts to link exposures to racial discrimination with morbidity experiences among minoritized individuals and communities. Studies could include efforts that elucidate interactions between racialized contexts in shaping health, such as specific life stages and settings for experiences of discrimination for discrete populations. Research could also include more sophisticated analyses of policies, such as redlining and resultant differences in built environments and health-promoting environments, which are associated with inequitable health outcomes. Regarding the former set of studies, examinations of how distinctive combinations of institutional policies of specific places reinforce social marginalization could help devise more robust tactics for pursuing equity with populations whose social positions are reinforced by particular racialization and multiple, overlapping minoritization processes. New discoveries here are key to overcome limitations of strategies for addressing racism through universal remediation — which ignore important within-population and between-population differences in structural positioning that can vary exposures to chronic stress and the availability of protective social and socioeconomic capital. In addition, actively considering intersections of multiple interlocking systems of privilege and oppression, such as racism, heterosexism, and cisgenderism, in shaping health allows health care and public health to be in a better position to address the compounded effects of these systems on physical and mental health (16,18,31,47–50). Each new effort here advances use of intersectional frameworks that give increasingly more relevant service to populations whose social positions relative to well-being are jointly determined by the many social systems, processes, and hierarchies stratifying society. Regarding the latter category of studies, analyses of interdependencies in nested policy hierarchies and networks governing racial equity and evidence-driven recommendations for altering them are crucial to demolish racist systems effectively and permanently. Better addressing enmeshed local, state and territorial, and national policies linked to racially disparate treatment and disproportionate impact could clear grounds upon which antiracist systems could be constructed.

The articles in this collection also highlight the need for educational institutions in medicine and public health to look within themselves to identify and dismantle fundamentally racist norms, pedagogies, and processes that perpetuate racist practices in clinical and public health practice and research. Efforts should examine and reform admissions and hiring practices, curricula, teaching and mentor training and hiring practices, approaches to retain and promote minoritized individuals and staff, and institutional partnerships and contracting practices. Strategies for effectively synergizing organizational change efforts of individual institutions to eliminate systemic racism require additional attention. Strides here are key to transforming racism initiatives within institutions into movements capable of tackling racism in health care and public health systems.

Increased attention is also needed to codify and actualize the imperative of meaningfully engaging community partners in focused efforts to address inequities in housing, food insecurity and poverty, and other “nonhealth” domains that affect health. We must find ways to make community-centered strategies that incorporate multisystemic, intersectional approaches our norm and mandate. Doing so may more effectively blend and leverage community and institutional assets, evidence, and know-how to address racism in all systems affecting health opportunity.

Additional gaps in ongoing work, not highlighted in this collection, should also be addressed. For example, the public health sector should become more actively engaged in efforts to dismantle policy-mediated causes of racial health inequities. Novel strategies, including partnerships with grassroots action efforts (ie, emanating from communities) that inform system changes could be pursued to stimulate action to develop or support implementation of antiracist policies. Similar strategic partnerships with other nonhealth sectors (eg, business, justice) for maximum effectiveness could create powerful alliances with the potential to influence social change in and across systems linked to racial and ethnic differences in health. Within the public health sector itself, work to synchronize and achieve strategic alignments of antiracist interventions in the areas of education, research, and public health practice will amplify and accelerate progress toward inseparable racial and health equity goals.

Lastly, further examination of the role of the COVID-19 pandemic in reinforcing the very systemic racism responsible for observed disproportionate burden of COVID-19 among some racial and ethnic minority populations should be contemplated as directions for future effort are considered. New longitudinal efforts here could describe and address the long-term consequences of systemic racism not only for the patterning of COVID-19 health disparities by race and ethnicity but also for the persistence and patterning of chronic disease disparities and inequities in the social determinants of health. These efforts could cover potential additional increases in morbidity and mortality that may occur among some racial and ethnic populations as COVID-19 becomes endemic. But they might also cover possible enduring functional limitations and chronic conditions that could be associated with complications of COVID-19, such as the development of 1) multisystem inflammatory syndrome in children, 2) multisystem inflammatory syndrome in adults, and 3) post-COVID-19 conditions (also known as long-COVID and postacute sequelae of SARS-CoV-2 infection). Additional variations in subsequent disproportionate effects by race and ethnicity on well-being for different age cohorts should also be explored. This exploration is suggested because the consequences of the COVID-19 pandemic for social development, social participation, social network density, and psychosocial resource availability likely also vary by chronological age and social placement during the life course within race and ethnicity. Lastly, the disparate systemic implications of COVID-19 for the socioeconomic positions, collective efficacy, and access of vital conditions for health and well-being (eg, humane housing, quality education, meaningful work and wealth) of racially and ethnically diverse communities should be continuously documented and addressed. Doing so may ensure that the increased attention to systemic linkages between racism, health, and well-being stimulated by COVID-19 and social injustices occurring during the past 3 years will be sustained and leveraged toward societal transformation.

At a health systems level, pandemic-associated racial and ethnic inequities in access to prevention and treatment should be further dissected and prospectively monitored. First, health systems research could continue to identify circumstances where access inequities existing before the onset of the COVID-19 pandemic may have been exacerbated (eg, access to screenings, treatments, or procedures for breast cancer) (51). Characterizing and addressing the differential effects of such access inequities on population health care trajectories across time is essential to prevent further expansion of health gaps that widened during the pandemic. Second, equitable receipt of COVID-19 vaccines, novel therapeutics (eg, monoclonal antibody therapies and oral antiviral therapeutics), and expedited treatment of individuals who received a positive test result for the virus remain essential to reduce disparities in severe COVID-19–associated illness and deaths that continue to affect some racial and ethnic minority populations (52–54). Accordingly, research that clarifies strong leverage points and tactics for severing pathways through which structural racism shapes inequities in access to such modalities among racially and ethnically diverse populations is important to improve enjoyment of the protective benefits of these interventions by people with higher risks for exposure to SARS-CoV-2 and for adverse outcomes. Moreover, evaluations of supply prioritization, allocation, and distribution strategies and resource triage protocols enacted during the pandemic may provide evidence that strengthens the case for giving precedence to racial equity considerations when deciding how to deploy scare resources as SARS-CoV-2 continues to evolve (54,55). Work in these 2 highlighted areas could secure health system changes that ensure all persons have fair and just opportunities to avoid, cope with, and recover from the effects of COVID-19, regardless of their race or ethnicity.

Ultimately, work to dismantle racist systems present in health will require multipronged efforts that draw on numerous strengths from within and outside health care and public health institutions. As this work moves forward, our fields are called to consider bold and innovative actions that have the potential to produce lasting change.
